# The Microstructural Reconstruction of Variously Sintered Ni-SDC Cermets Using Focused Ion Beam Scanning Electron Microscopy Nanotomography

**DOI:** 10.3390/ma17133068

**Published:** 2024-06-21

**Authors:** Gregor Kapun, Endre Majorovits, Sašo Šturm, Marjan Marinšek, Tina Skalar

**Affiliations:** 1 National Institute of Chemistry, Hajdrihova 19, 1001 Ljubljana, Slovenia; gregor.kapun@ki.si; 2 Carl Zeiss Microscopy GmbH, ZEISS Gruppe, Carl-Zeiss-Strasse 22, 73447 Oberkochen, Germany; endre.majorovits@zeiss.com; 3 Jožef Stefan Institute, Jamova 39, 1000 Ljubljana, Slovenia; saso.sturm@ijs.si; 4 Jožef Stefan International Postgraduate School, Jožef Stefan Institute, Jamova Cesta 39, 1000 Ljubljana, Slovenia; 5 Faculty of Natural Sciences and Engineering, University of Ljubljana, Aškerčeva Cesta 12, 1000 Ljubljana, Slovenia; 6 Faculty of Chemistry and Chemical Technology, University of Ljubljana, Večna pot 113, 1000 Ljubljana, Slovenia; marjan.marinsek@fkkt.uni-lj.si

**Keywords:** solid oxide fuel cells, cermets, focused ion beam scanning electron microscopy, microstructure, morphology

## Abstract

This work focuses in-depth on the quantitative relationships between primary first-order microstructural parameters (i.e., volume fractions of various phases and particle size distribution) with the more complex second-order topological features (i.e., connectivity of phases, three-phase boundary length (TPB_L_), interfacial areas, or tortuosity). As a suitable model material, a cermet nickel/samaria-doped ceria (Ni-SDC) is used as an anode in a solid oxide fuel cell (SOFC). A microstructure description of nano-sized Ni-SDC cermets, fabricated at various sintering conditions from 1100 °C to 1400 °C, was performed using FIB-SEM nanotomography. The samples were serially sectioned employing a fully automated slicing procedure with active drift correction algorithms and an auto-focusing routine to obtain a series of low-loss BSE images. Advanced image processing algorithms were developed and applied directly to image data volume. The microstructural–topological relationships are crucial for the microstructure optimisation and, thus, the improvement of the corresponding electrode performance. Since all grains of individual phases (Ni, SDC, or pores) did not percolate, special attention was given to the visualisation of the so-called active TPB_L_. Based on the determined microstructure characteristics of the prepared Ni-SDC cermets, including simulations of gas flow and pressure drop, thermal treatment at 1200 °C was recognised as the most appropriate sintering temperature.

## 1. Introduction

Modern solid oxide fuel cells (SOFCs), perspective devices for clean and efficient energy conversion, operate at temperatures between 600 and 800 °C. Yttrium-stabilized zirconia (YSZ) as a ceramic part of a cermet is normally replaced with ionically superiorly conductive samarium (SDC) or gadolinium-doped ceria (GDC) [1,2]. Specifically, at comparable temperatures, GDC or SDC exhibit ionic conductivities of nearly one order of magnitude greater than YSZ [3,4,5,6,7]. Since the anode side in a SOFC is the area where the oxidation of fuel gas occurs, an anode material must fulfil several important requirements: (i) it has to be highly catalytically active for the oxidation of the fuel gas, which is determined with the number of reaction sites; (ii) the anode has to exhibit chemical, morphological, and dimensional stability at the required operating temperatures in the reductive fuel gas environment; (iii) the anode material has to exhibit the greatest possible electronic- and ionic-conductivity to minimise ohmic losses and to promote the reaction of the fuel with oxide ions; (iv) it also has to exhibit chemical, mechanical, and thermal compatibility with adjacent cell components; and (v) the porosity of the anode must be modified to optimise the mass transport of fuel or gaseous product components. Many of these requirements are strongly determined by the anode microstructural features, such as electrode composition, particle size distribution, phase connectivity, tortuosity, and triple phase boundary length [8,9,10,11,12]. In challenges to find appropriate correlations between the anode microstructure and selected measured properties, researchers frequently rely on 2D cross-sectional images obtained from optical and/or electron microscopy [13]. However, the main problem associated with the processing of 2D images and obtaining essential microstructural features is that 3D microstructural characteristics are estimated from 2D images using porous models based on geometrical theories: general effective media (GEM) [14,15], the contiguity concept [16], the random register network model [17], the random packing spheres model [18], and stochastic reconstruction [19]. All the above-mentioned models are based on various assumptions, which can only indirectly explain the real 3D microstructure. In this context, recent progress in high-resolution tomography utilizing a focused ion beam (FIB) has opened new analytical possibilities and, thus, initiated new potentials for the microstructure–performance relationship investigations in composite SOFC anodes [9,20,21,22,23,24,25,26]. Through such real 3D measurements, essential microstructural parameters, such as three-phase boundary length (TPBL) and tortuosity, can be obtained. The FIB tomography approach consists of ablating a structure physically by FIB cutting, followed by a digital reconstruction based on SEM images taken after each ablation step. State-of-the-art FIB-SEM tomography can reduce voxel dimensions down to 3 × 3 × 3 nm^3^ while the size of the analysed volume can still remain in the range of 10 × 10 × 10 μm^3^ due to the automated process [27,28,29]. Such precise volume analysis offers very accurate 3D microstructural information in areas still large enough to provide a general depiction of the material. The reconstructed 3D microstructure can then be either analysed to extract 3D microstructural parameters or directly applied to numerical models in order to calculate some transport phenomena or electrochemical performance of the corresponding material [30,31,32,33,34,35,36,37,38]. The 3D microstructural information can be divided into so-called first-order parameters (including electrode composition and particle sizes), which are normally tuneable during materials processing and second-order parameters (including connectivity of phases, TPBL, interfacial areas, or tortuosity), which can be directly linked with the electrode electrochemical performance [39,40,41]. Among the second-order parameters, special attention is normally given to the quantisation of the TPBL [42,43]. The TPB points are the geometrical site where the ion-conducting ceramic phase (i.e., SDC or GDC) meets with the electronically conductive metal phase (Ni) and the gas-transporting pores, thus enabling fuel oxidation electrochemical reaction. However, the TPB is only active when all three phases (ceramic, metallic, and pores) are connected to their respective base. For this reason, the connectivity aspects must also be considered in order to distinguish between active and inactive TPBL and properly correlate material microstructure with its electrochemical activity. In general, the overall TPBL is normally much higher if a finer-grained anode cermet is applied when compared to coarser-grained cermets [44,45]. However, a preferable finer-grained cermet can be achieved if lower sintering temperatures are applied during electrode preparation, which, in contrast, may be the origin of somewhat lower phase connectivity. Therefore, the determination of 3D microstructural parameters must undergo in-depth analysis in order to extract crucial microstructural information, which could be used to establish a quantitative relationship between first-order microstructural parameters that can be altered during material processing with the second-order topological parameters that define the electrochemical performance of the anode.

In the present study, an optimised FIB-SEM with advanced image processing and 3D watershed segmentation was used in order to obtain a high-quality 3D reconstruction of Ni-SDC anodes. Several chemically identical but microstructurally different Ni-SDC cermets were prepared by varying sintering temperatures. Subsequently, from collected 3D data, several microstructural parameters (i.e., volume fractions, phase connectivity) and interfacial parameters (i.e., interface area, active TPBL) for each cermet were quantified. Additionally, tortuosity was estimated by solving the diffusive transport equation of the analysed volumes. Basic microstructural relationships between primary parameters and higher-order topology were discussed in detail.

## 2. Materials and Methods

### 2.1. Sample Prepaartion

Ni-SDC preparation via the citrate–nitrate combustion synthesis started by mixing aqueous solutions of appropriate metal nitrates and citric acid. Cation precursors (i.e., hydrated metal nitrates Ce(NO_3_)_3_·6H_2_O (99%, Sigma-Aldrich, St. Louis, MO, USA), Sm(NO_3_)_3_·6H_2_O (99.9%, Alfa Aesar, Haverhill, MA, USA), and Ni(NO_3_)_2_·6H_2_O (99%, Acros Organics, Geel, Belgium) and citric acid (100%, Carlo Erba, Cornaredo, Italy)) were separately dissolved in distilled water. The mixture was prepared so as to ensure the final molar ratio between cerium and samarium cations in SDC to be 80:20, considering that the Ni and SDC mass contents in the final solid composite were 50%. In order to ensure a subsequent self-sustaining combustion reaction, the molar ratio between citric acid and nitrates was set to 0.2. The prepared aqueous mixture was then treated over a water bath at 60 °C under vacuum (7 mbar) for 6–7 h until it transformed into a light green brittle solid gel. The dried solid was ground and compressed into pellets (*ϕ* = 12 mm, *h* = 30 mm, *p* = 100 MPa), which were, afterwards, immediately ignited at the top using a hot metal tip to initiate a self-sustaining combustion reaction that propagated as a reaction zone throughout the pellet. In order to eliminate agglomerates, the synthesised NiO-SDC residue was next crushed in an agate mortar, wet-milled (isopropanol) in a planetary mill (Fritsch pulverisette 7 premium line) for 15 min with 10 mm grinding balls and 500 rpm (1st milling) and additionally 15 min with 1 mm grinding balls and 500 rpm (2nd milling). After subsequent drying, the milled NiO-SDC powder was calcined at 800 °C for 1 h and then uni-axially pressed into cylinders (*ϕ* = 6 mm, *h* = 2.6 mm, *p* = 100 MPa). The formed cylinders were sintered at various temperatures ranging from 1100 °C to 1400 °C for 1 h and reduced in an Ar-H_2_ (5 vol%) atmosphere at 900 °C for 2 h. Prior to the 2D microstructure determination of the sintered NiO-SDC composites, all tablets were polished and thermally etched.

### 2.2. Characterization Techniques

The rapid temperature changes during citrate–nitrate combustion within the reaction zone were measured as a temperature profile using Impac IPE 140, Advanced Energy, Denver, CO, USA, optical pyrometer based on sample brightness. The measuring range of the pyrometer is from 50 to 1200 °C; it has a very quick response time (1.5 ms) and an accuracy of ±2.5 °C below 400 °C and ±0.4% of a measured value above 400 °C. Particle size observation of the as-synthesized NiO-SDC nano-dispersion was conducted on a Cs-corrected JEOL ARM 200F, Jeol, Tokyo, Japan, transmission electron microscope (TEM) operated at 200 kV and equipped with EDXS. Samples for TEM were prepared by dispersing the obtained NiO-SDC powder in ethanol via ultrasonication and subsequent deposition of the suspension on holey carbon-coated copper grids. A Microtrac Bluewave, Microtrac Inc., Montgomeryville and York, PA, USA particle sizer was used to determine particle size distributions (measured as aqueous suspensions) of unmilled and milled NiO-SDC powders after the synthesis. A heating microscope (Leitz Wetzlar, Wetzlar, Germany) was used to obtain the shrinkage curve of NiO-SDC during sintering up to 1450 °C with a constant heating rate of 10 K min^−1^. Conventional 2D cross-section imaging of sintered and reduced Ni-SDC pellets was conducted using FE-SEM (Zeiss, Ultra Plus, Zeiss, Oberkochen, Germany). FIB-SEM (Zeiss, Crossbeam 540, Zeiss, Oberkochen, Germany) was employed for image data stack acquisition of sintered and reduced anode material to obtain a 3D reconstruction of the probed volume.

### 2.3. Microstructural Measurements

#### 2.3.1. FIB-SEM Sample Preparation

For FIB-SEM microscopy, Ni/SDC samples after the reduction were fractured and infiltrated with low-viscosity resin (Epofix, Struers, Copenhagen, Dennmark) in a controlled vacuum regime, slowly ramped down to 200 Pa, and then slowly ramped up to atmospheric pressure (EpoVac, Struers, Copenhagen, Dennmark) so that an improved contrast could be generated between pore and solid phases during subsequent SEM imaging after each FIB ablation step. The impregnated samples were then cured at atmospheric pressure and room temperature for 48 h.

After resin infiltration, samples were precisely cut using a 150 μm slicing step (Wire Saw Solutions Group LLC, Bethlehem, PA, USA). The obtained thick lamellas were attached to cylindrical stubs and mechanically polished to obtain lamellas with a final thickness of ~30 μm. The thinned Ni-SDC lamellas were carefully attached under a stereo microscope to the edge of the Al stub using Ag-conducting cement and sputtered with a 100 nm platinum conductive layer (Figure S1).

#### 2.3.2. FIB-SEM Imaging

FIB-SEM imaging was performed using a Zeiss Cross-beam 540 microscope, Zeiss, Oberkochen, Germany equipped with 3D Atlas software v5.1, Zeiss, Oberkochen, Germany. The FIB region of interest (ROI) preparation can be summarised in the following steps: (i) at a representative cross-sectional area of the sample, 1 μm of Pt protection layer was initially deposited using a metalloorganic-ion source; (ii) subsequently, two lines converging at an angle of 40 degrees from the outside edge and three parallel central lines were shortly milled using FIB gun; (iii) three rectangular carbon bands with final thickness of 100 nm were deposited on top of the pre-milled lines using organic-ion source; (iv) finally, the as-prepared high contrast fiducial markers were sandwich protected by repeating the same Pt deposition procedure as in initial step (Figure S2). After front trench removal by FIB milling, the special carbon reference markers could be clearly distinguished due to the high Z-contrast difference compared to the Pt layer. The new approach, with embedded fiducial markers within the protection layer, enables very precise reference marker tracking at high-resolution SEM imaging conditions.

**Figure 1:**
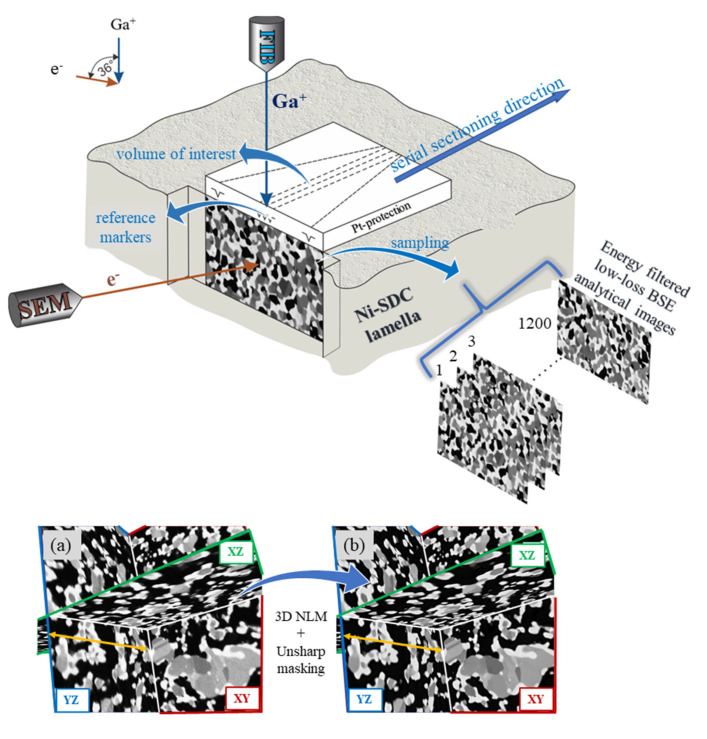
FIB-SEM imaging set-up (above), visualization of 3D image data volume using orthogonal planes: (**a**) raw image data volume; (**b**) image data volume after direct 3D image processing. Half orthogonal plane dimension, marked with the yellow arrow on the YZ plane, corresponds to 1.5 mm (below).

The samples were then serially sectioned using a fully automated slicing procedure with advanced drift correction algorithms and an auto-focusing routine to obtain a series of 2D images (LL-BSE images) with narrow and reproducible slice thickness (Figure 1). The SEM imaging parameters were optimised at HV 1.5 kV, probe current 2 nA, and acquired at an angle of 40° with the use of dynamical focusing and real-time tilt corrections.

### 2.4. Three-Dimensional Data Processing

#### 2.4.1. Three-Dimensional Image Processing

The raw image stack was imported into Amira 2020.2 software, Thermo Fisher Scientific, Hillsboro, Oregon, USA and post-process realigned using the least-square method. State-of-the-art image processing was used by applying a 3D Non-Local Means (3D NLM) filter directly on the raw 3D image data volume. Afterwards, an unsharp masking filter was used to reinforce the contrast at the edges and achieve a sharper appearance of the details (Figure S3). In this manner, very effective smoothing was achieved while the phase contrast and grain edges were both successfully preserved. In each case, a high-quality 3D image data volume that reflected an actual Ni-SDC microstructure was obtained.

#### 2.4.2. Segmentation and 3D Reconstruction

**Figure 2:**
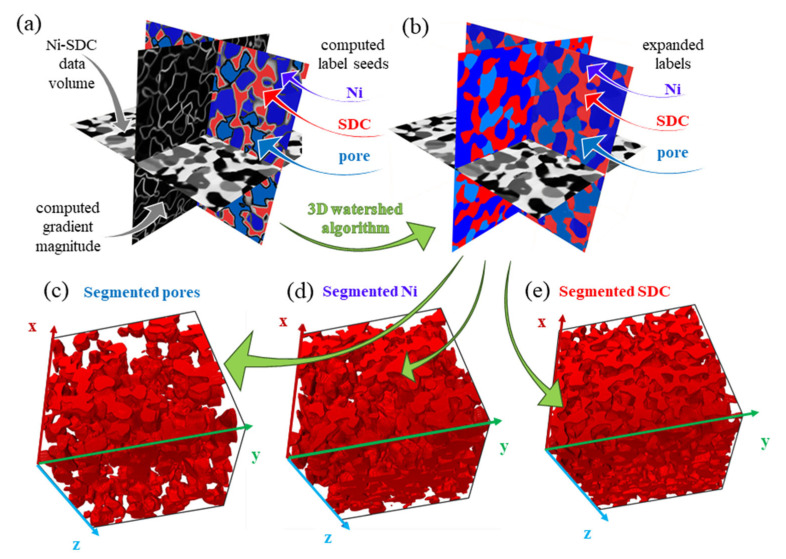
Basic 3DWS procedure: (**a**) data input prior to expansion step; (**b**) computed 3D watershed transformation; (**c**) segmented pore volume; (**d**) segmented Ni volume; (**e**) segmented SDC volume.

The 3D image data volumes were segmented using an advanced 3D watershed segmentation (3DWS) procedure. The developed 3DWS procedure includes two major steps: (i) an initial classification of some voxels into two or more phases to generate so-called label seeds (described in [46], followed by (ii) an expansion step where those seeds are expanded until all voxels are labelled. The expansion of label seeds was performed three-dimensionally using the watershed algorithm until all voxels were assigned to pore, Ni, or SDC phases (Figure 2).

### 2.5. Three-Dimensional Quantification of Porous Microstructure

#### 2.5.1. Volume Fractions

The volume fraction of each phase was calculated by counting the voxel elements corresponding to individual phase *N_i_* and divided by the total number of voxels *N_tot_* within the analysed cuboid using Equation (1):

(1) $$\varphi_{i}=\frac{N_{i}}{N_{tot}}=\frac{V_{i}}{V_{tot}}$$

#### 2.5.2. Size Distribution

After 3DWS and phase separation, individual objects within continuously connected solid phases were separated in accordance with the procedure described in the literature [47,48] by using the following procedure (Supplementary Figure S3): (i) the extracted phase (Supplementary Figure S3a) was used for the calculation of distance function of a binary image onto a grey level image as the sum of successive erosions (Supplementary Figure S3b); (ii) individual particles were marked based on regional maxima calculation and separated using the watershed algorithm (Supplementary Figure S3c); (iii) after connectivity analysis of individual objects within the entire 3D volume, each separated particle was assigned to a different consecutive value (Supplementary Figure S3d). By using the above procedure, both solid phases within examined Ni-SDC cermets were successfully three-dimensionally separated from the continuous phase (Supplementary Figure S3e) into individual particles (Supplementary Figure S3f), which were used in quantitative measurements using feret3D analysis. Quantification was performed by including at least 9000 particles.

#### 2.5.3. Specific Surface and Specific Interfacial Areas

The specific surface area (*SSA*) and specific interface area (SIA) of the complex porous materials were obtained directly from the 3D-reconstructed surface described with triangular approximation by using Amira 3D 2020.2 processing software, Thermo Fisher Scientific, Hillsboro, OR, USA and the marching cubes algorithm in several iterations as described in [49]. When the surface area of individual phase *S_i_* is divided by the total volume of analysed cuboid *V_tot_*, an *SSA* is obtained according to Equation (2).

(2) $$SSA=\frac{S_{i}}{V_{tot}}$$

The interfacial specific area (SIA) reveals the surface, which is shared between two adjacent phases and can be obtained by solving the following system of linear equations (for Ni-SDC cermet: *n* = 3):

(3) $$A_{phase\;1}=A_{\frac{phase\_1}{phase\_1}}+A_{\frac{phase\_1}{phase\_2}}+\ldots +A_{\frac{phase\_1}{phase\_n}}\;A_{phase\;2}=A_{\frac{phase\_2}{phase\_1}}+A_{\frac{phase\_2}{phase\_2}}+\ldots +A_{\frac{phase\_2}{phase\_n}}\;\ldots \;A_{phase\;n}=A_{\frac{phase\_n}{phase\_1}}+A_{\frac{phase\_n}{phase\_2}}+\ldots +A_{\frac{phase\_n}{phase\_n}}$$

#### 2.5.4. Phase Connectivity

The connectivity analysis of the reconstructed microstructure was performed by using a cluster neighbourhood rule [50]. The two voxels were judged to be connected only if they were in full contact by sharing a common face. More specifically, they were considered not to be connected when the adjacent voxels were in contact only by a vertex or an edge. In this study, the individual phase was defined as continuous only when the corresponding voxels were continuously connected to the six boundaries of the reconstructed cuboid, while the other portion of the phase was considered isolated.

#### 2.5.5. TPB Calculations

There are several approaches to quantifying TPBL, ranging from the simple voxel edge counting [44] to the centroid smoothed edge-counting [9] and the volume expansion method [34], to name just a few. Although the quantitative predictions of these methods differ due to differences in the underlying computational schemes, they are useful for the comparison of relative performances of different materials. Here, we designed and applied an algorithm that first identifies all TPB elements/pixels on the segmented 3D grid and then searches for the closest and smoothest connecting paths between them. The TPB elements were searched only within the gas/void phase elements that had the other two-phase types among the nearest elements. Using a distance criterion, we selected groups of three element points to determine their centroid. The line connecting the centroid points was selected to contribute to the TPB path if it was shorter than the body diagonal of the cubic volume element.

#### 2.5.6. Tortuosity

Accurately determining tortuosity from the pore structure is crucial for flow behaviour simulation due to its opposite effect on permeability [51]. In the present study, the tortuosity is determined as a geometric parameter *τ*; it is defined as the ratio between the average length of the actual fluid path (*L_a_*) and the nominal path length (*L*) (Equation (4)):

(4) $$\tau =\frac{L_{a}}{L}$$

For *L_a_* calculation through the pore phase, the Amira 3D 2020.2 processing software, Thermo Fisher Scientific, Hillsboro, OR, USA was used, with which the centroid of every plane fracture and path length through them were computed. The final value was divided by the number of planes along the flow direction, as described in the literature [52].

#### 2.5.7. Absolute Permeability

**Figure 3:**
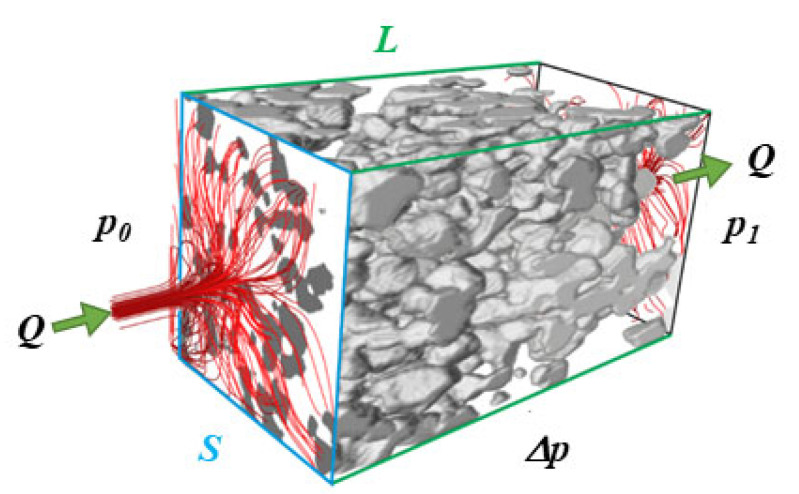
Schematic presentation of the absolute permeability simulation concept of the fuel gas transport throughout the Ni-SDC porous network where *Q* is the global flow rate, *S* is the cross-section of the porous medium, and *L* is the length of the sample in the flow direction.

The advanced 3D data processing of the porous phase enables the possibility of gas flow and permeability simulation throughout porous Ni-SDC cermet. For the case of a simple homogenous porous network, the computational results for gas flow resistance could be expressed by following Darcy’s law [53]. In the present work, the absolute permeability (AP) experiment was performed in Avizo 2020.2 software, Thermo Fisher Scientific, Hillsboro, OR, USA (with XLab extension) by solving Darcy’s law in the simulation of fuel gas flow throughout a three-dimensional porous network model of the corresponding Ni-SDC cermet. Figure 3 schematically presents an AP simulation experiment in which an extracted porous network phase is hermetically closed on four faces while experimental setups are added on two opposite faces to guide the gas flow along one direction. 

The AP experiment was simulated throughout a 3.2 μm × 3.2 μm cross-sectional porous network area along the *L* distance of 3.2 μm with the pressure difference Δ*p* = *p*_0_ − *p*_1_ = 30 kPa.

## 3. Results and Discussion

**Figure 4:**
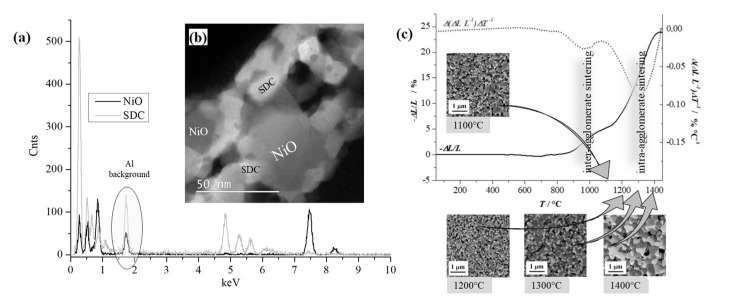
(**a**) HAADF STEM image of NiO-SDC mixture after the synthesis (**b**) HR HAADF STEM image with EDXS spectra positions of SDC and NiO individual grains and (**c**) sintering behaviour of NiO-SDC composite calcined at 800 °C (full line) and derivative sintering curve (dashed line) with SEM micrographs of NiO-SDC mixture at selected temperatures.

The main advantages of combustion-derived NiO-SDC synthesis are its relative simplicity and very quick material synthesis. The utilised citrate–nitrate combustion synthesis is a single-step method with rapid temperature change within the citrate–nitrate combustion zone. Typically, during the NiO-SDC synthesis, the velocity of the reaction propagation wave is 0.6 mm s^−1^, while the temperature gradient in the head of the combustion zone is calculated as ~1050 °C s^−1^. Due to the relatively large amount of heat released during the synthesis, the measured peak combustion temperature is 1169.2 °C. However, during the synthesis, the system is exposed to relatively high temperatures for a rather short period of time. The temperature rise from room temperature to the combustion temperature happens in a fraction of a second, while after the combustion reaction, the residue ash is also quickly cooled. Such extreme temperature variations within a significantly limited period, during which diffusion in a solid system plays a significant role, typically lead to a nano-scaled oxide mixture of NiO and SDC (Figure 4a). The mixture is random, and the observed nanoparticles can be considered single-phase regions (SDC or NiO) as determined by the EDXS analysis and electron diffraction (Figure 4b, Figures S5 and S6). The average grain sizes of NiO and SDC phases after the synthesis, as determined from TEM images, are 17.3 nm and 8.6 nm, respectively. After the synthesis, these NiO and SDC grains are partially agglomerated. It appears that there are two levels of agglomeration, with an average agglomerate size of 3.3 μm and 82 μm for the first and second agglomeration levels, respectively (Figure S7). However, the formed agglomerates of NiO-SDC are rather weak and can be easily reduced in size to ~400 nm after the first milling cycle and <100 nm after the second milling cycle.

The nano-sized NiO-SDC mixture sinters relatively well (Figure 4c). During sintering, the material’s microstructure gradually develops. Shrinkage starts at ~800 °C and is practically completed at ~1400 °C with a final shrinkage of ~24% (estimated sintering temperature 1300 °C). The densification of the material proceeds over two separate steps. Consecutive sintering steps are a consequence of the previously mentioned partial agglomeration of the as-synthesized powder. The first densification step with the maximal sintering rate at 950 °C may be referred to as inter-agglomerate sintering and is an effect of the highly sinterable nano-sized oxide mixture. The second densification step (intra-agglomerate sintering) starts at rather higher temperatures (above ~1100 °C), reaching the maximal sintering rate at ~1300 °C. According to SEM images within Figure 4c, the grains of both phases grow, and porosity decreases with increased sintering temperature. Nevertheless, sintering up to 1400 °C still keeps grains of both phases well within the sub-micrometre range.

**Figure 5:**
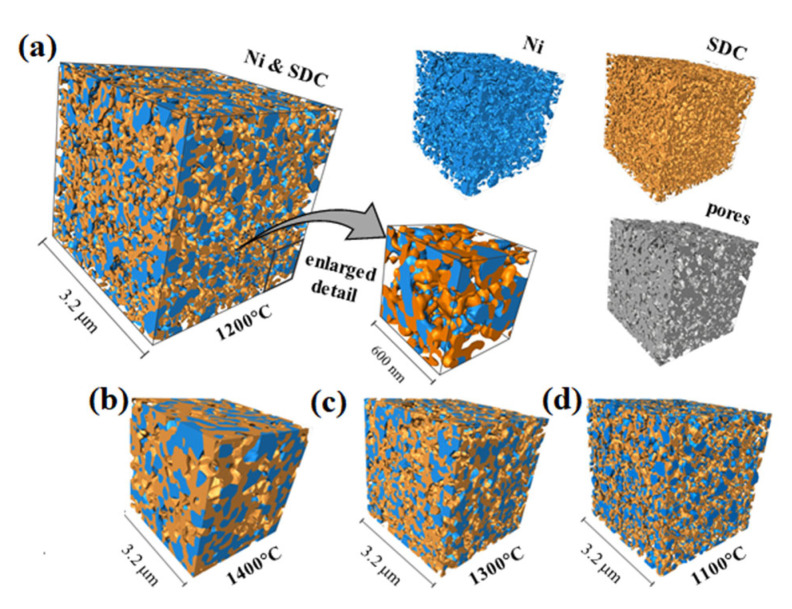
Three-dimensional reconstructed Ni-SDC cermets sintered at various temperatures (Ni: blue, SDC: orange, pores: transparent or in image showing extracted pores: grey): (**a**) 1200 °C, (**b**) 1400 °C, (**c**) 1300 °C, and (**d**) 1100 °C. Owing to comparison issues, the presented reconstructed cuboids are cropped to the same volume.

NiO-SDC sintering should provide good contact between particles within each phase (NiO or SDC) and between both phases in order to adjust important primary microstructural parameters properly. Since a reasonable evaluation between microstructure and anode electrochemical performance can be deduced only from a 3D microstructure analysis, a series of volumes reconstructed with FIB tomography of the graded Ni-SDC cermets after the reduction are shown in Figure 5. Due to relatively large analysed volumes and a sufficiently small voxel size (Table 1), it is assumed that in each case, the FIB data illustrate a representative volume of the prepared Ni-SDC mixture. Furthermore, the average particle-size-to-voxel-size ratio is believed to be large enough to image the particles accurately and, thereby, obtain small errors in the microstructural parameters derived from the data. As FIB microstructure analysis strongly depends on reliable object recognition, the three continuously networked percolating phases (Ni, SDC, and pores) are split into discrete objects with an advanced method using direct 3D image processing and a special 3D watershed segmentation algorithm developed for this purpose. The volume ratio of phases in the prepared cermets (vol.% Ni/vol.% SDC), calculated from 3D data, is determined as 0.83, 0.82, 0.81, and 0.83 for cermets sintered at 1100 °C, 1200 °C, 1300 °C, and 1400 °C, respectively, which agree well with the expected values 0.82 (within ±0.01% error). The determined vol.% Ni/vol.% SDC ratios for all four samples are very similar, indicating that reconstructed volumes, indeed, represent average samples in all cases.

Some first-order microstructural parameters of the prepared Ni-SDC composites are summarised in Table 1. Parameters *feret-x*, *-y*, *-z*, and $\bar{d}$ re represented as intercept lengths in the *x*, *y*, and *z* directions, respectively, and as feret-3D while porosity *ε* is determined as microstructural porosity and shape factor *Ψ_W_* as sphericity defined by Wadell. For statistically reliable microstructure characterisation, the total reconstructed volume in each case included at least 10^4^ particles of a respective phase. In general, after the reduction, the porosity decreases, and the grains of each phase grow with the increasing sintering temperature. The final microstructural porosities are determined as 39.7%, 35.6%, 29.3%, and 18.9% if samples are sintered at 1100 °C, 1200 °C, 1300 °C, or 1400 °C, respectively. At the same time, a rather interesting phenomenon is observed regarding the average sizes of the two solid phases (Ni or SDC). Specifically, it is very important that a rigid ceramic framework is established during sintering, which should prevent exaggerated Ni grain growth during subsequent reduction and keep Ni grains within the sub-micrometre range. The latter is important since only relatively small grains of both solid phases could ensure a large TPB length in the final anode microstructure. However, the expected tendency of Ni-grain growth with increasing sintering temperature is valid for sintering temperatures above 1200 °C. Sintering temperature as low as 1100 °C surprisingly results in relatively large Ni grains in the final Ni-SDC cermet (1.64 times larger than at 1200 °C). Due to the low sintering temperature, these relatively large Ni-grains cannot be a consequence of exaggerated NiO sintering. In contrast, due to the early stage, the sintered grains of both phases are still relatively small and apparently do not form a continuous rigid 3D structure because intra-agglomerate sintering has not started yet. Therefore, enlarged Ni grains must form during the NiO to Ni reduction and subsequent sintering of individual Ni grains. The driving force for this Ni-phase grain growth is a decrease in the interface energy between Ni and SDC. Another consequence of the not-rigid SDC framework must be that after sintering at 1100 °C, a relatively large number of SDC grains is still not interconnected and, thus, does not form a continuous phase. Such material cannot be dimensionally and morphologically stable at the required operating temperature under the fuel gas environment in an operating fuel cell. Grains of both phases are irregularly shaped. With respect to the shape factor, sintering above 1200 °C results in a slight decrease. In contrast, the excessive Ni growth after the sintering at 1100 °C and subsequent NiO-to-Ni reduction results in more irregularly shaped over-sintered Ni-grains, while SDC follows the mentioned tendency toward an increasing shape factor with a lower sintering temperature.

**Figure 6:**
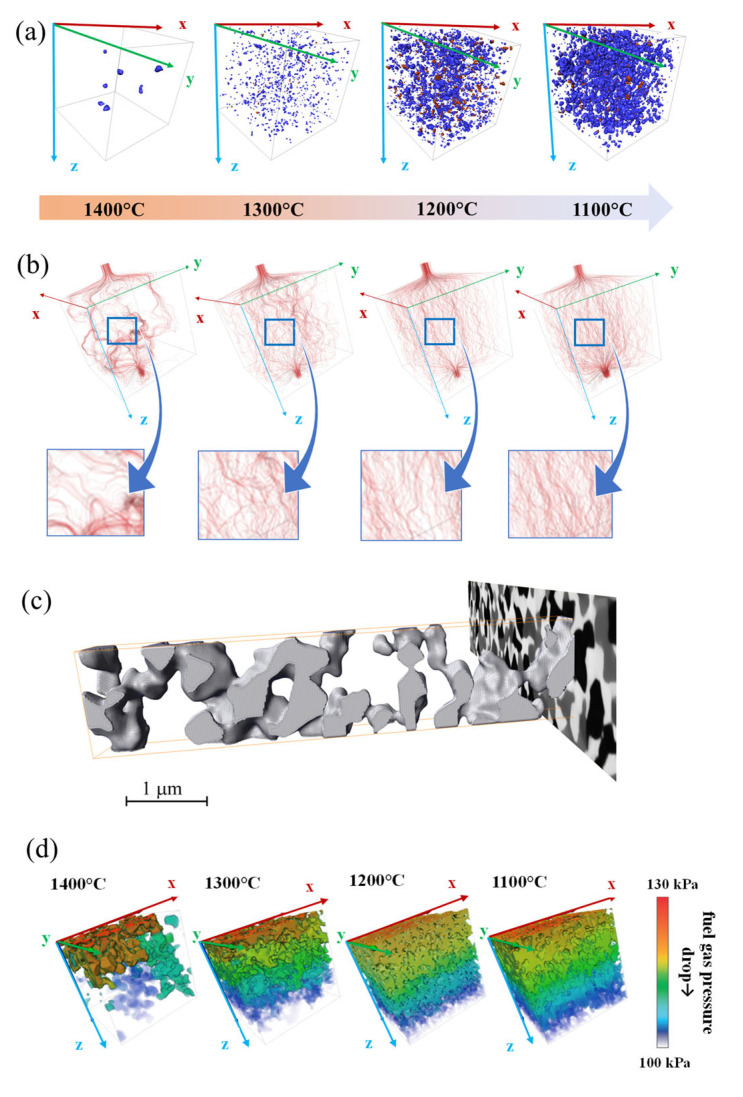
(**a**) Not connected phases (Ni and SDC); (**b**) permeability simulation of variously sintered Ni-SDC cermets with enlarged parts; (**c**) cutout of an individual pore in Ni-SDC cermet sintered at 1400 °C in z-direction and (**d**) three-dimensional visualisation of fuel gas pressure distribution in the Z direction of the porous network for the case of Ni-SDC cermet sintered at various temperatures. The edge of the cuboid corresponds to 3.2 μm for all cermets.

Even more informative than the first-order are the second-order microstructural parameters. Although Ni/SDC volume ratio remains the same in all four samples, variations in sintering temperature influence the volumetric connectivity of each phase. Volumetric connectivity, as one of the second-order microstructural parameters, is of prime importance regarding the ability of the material to transport gases in the pores as well as for the transport of charge carriers through solid phases. Only the connected phases can contribute to the electrochemical activity, while isolated parts are inactive. As expected, the connectivity of the two solid phases increases with increasing sintering temperature (Table 1). The solid phases practically form one large, percolated cluster if the applied sintering temperature exceeds 1300 °C. More precisely, the Ni percolated cluster represents 89.2% or 98.7% of the Ni volume if cermet is sintered at 1200 °C or 1300 °C, respectively. Similarly, the SDC percolated cluster represents 94.8% or 99.9% of the total SDC volume after being sintered at 1200 °C or 1300 °C, respectively. When a sintering temperature of 1100 °C is used, a relatively low connectivity value for the Ni phase (69.4%) can be observed (Figure 6a). Such low Ni connectivity is a consequence of the previously mentioned exaggerated Ni sintering after the NiO to Ni reduction. An opposite dependence on the temperature may be observed regarding pore connectivity. Specifically, the connectivity of pores decreases with sintering temperature from 99.8% to only 76.5% when increasing the sintering temperature from 1100 °C to 1400 °C. Such a decrease in porosity implies that pores must start to close due to solid phase sintering. Indeed, from the segmented FIB data, the overall porosity can be divided into opened and closed porosity (Table 1). It turns out that after sintering at 1400 °C, 4.5% of 18.9% of total porosity belongs to closed porosity. This 23.8% of total pore volume may no longer contribute to gas phase transport and is, thus, completely ineffective. In contrast, at a sintering temperature of 1100 °C, this close to overall porosity relation is only 0.2% to 39.7%. Even further, with advanced 3D data processing, pore distribution results can be used to simulate the permeability for gases through final cermets (Figure 6b). This simulation is performed with AvisoX-lab 2020.2 software, Thermo Fisher Scientific, Hillsboro, OR, USA in accordance with Darcy’s law. Interestingly, cermets sintered at temperatures of 1300 °C and, especially, 1400 °C contain relatively large regions that are practically impermeable to gases. This result further implies that such oversintered Ni-SDC cermets not only exhibit insufficient overall open porosity but are (regarding the same parameter) also locally inhomogeneous. Furthermore, since gas transport to and from active sites is necessary to prepare highly active cermet, the oversintered cermets must be locally completely inactive. In contrast, cermets sintered at 1200 °C and 1100 °C exhibit homogeneous pathway distribution for gas transport. In accordance with Darcy’s law [53], the ability of the prepared cermets to transmit a fluid is further expressed through their absolute permeability values k. The calculated values k, obtained simultaneously to permeability simulation in various samples with identically cropped areas of 3.2 μm × 3.2 μm × 3.2 μm, are 14.69 μm^2^, 37.56 μm^2^, 61.76 μm^2^, and 72.30 μm^2^ for cermets sintered at 1400 °C, 1300 °C, 1200 °C, or 1100 °C, respectively. As expected, the absolute permeability values decrease with increasing sintering temperature.

One additional fact can be deduced from the advanced 3D data processing. As shown in Figure 6c, the shape of an individual pore alongside one dimension can be visualised. It is evident that pores are irregularly shaped, implying that parameters such as an average pore diameter can be calculated but may have very little practical meaning. Namely, regardless of the sintering temperature, pores can locally have very small cross-sections, whilst, in a neighbouring region, they broaden practically into relatively large voids. The observed shape characteristic of pores can be expressed through the tortuosity factor *τ*. Tortuosity, as a parameter used to describe mass transport phenomena within a porous system, in general, is defined as the ratio of the effective average path of a fluid particle to the corresponding straight and shortest distance along the direction of macroscopic fluid [54]. The calculated τ values summarised in Table 2 are obtained in accordance with the centroid method and with respect to calculations of the average change in the location of the mean pore centroid between each 2D slice through the 3D dataset. To successfully employ this method, enhanced space resolution is required [45], which is assured through the relatively small voxel-size-to-analysed-volume ratio (128/216 nm^3^ μm^−3^, 128/216 nm^3^ μm^−3^, 250/512 nm^3^ μm^−3^, and 1000/2328 nm^3^ μm^−3^ for cermets sintered at 1100 °C, 1200 °C, 1300 °C, and 1400 °C, respectively). Furthermore, since tortuosity is a spatially oriented parameter, it is normally present in three cartesian axes. According to Table 2, the obtained *τ* values for all three phases are practically identical in various directions, pointing to an isotropic nature of the synthesised cermets, and are similar to some reports of other authors [33,37]. As expected, the sintering temperature affects the calculated *τ* values. More precisely, higher sintering temperatures increase the calculated *τ* values for the pore phase and decrease the calculated *τ* values for Ni and SDC phases. The calculated *τ* values for the pore phase are in good agreement with the obtained porosity and permeability results. A more open porosity after lowering the sintering temperature also means more direct pathways for gas transport. In contrast, the connectivity of solid phases after sintering and the subsequent reduction is the prime reason affecting the *τ* values. Lower Ni and SDC phase connectivity after lowering the sintering temperatures causes relatively larger *τ* values. This phenomenon is particularly evident in the case of cermet sintered at 1100 °C, for which the calculated *τ* value for the Ni phase increases substantially. Such a significant *τ* value increase points to some percolation problems in the Ni phase.

The above-discussed results regarding the porosity, permeability, and tortuosity of the prepared Ni-SDC cermets are somehow also reflected through the gas pressure distribution throughout Ni-SDC cermet during constant fuel flow rate. Figure 6d shows a visualisation of fuel gas pressure contours in the Z-direction within a porous network of the prepared cermets. From the results, it is evident that the fuel gas flow is not equally pressurised during transition along the Z axis by forming locally inhomogeneous pressure zones. The cause of that is related to inhomogeneous pore distribution in which pores can locally have very small cross-sections, while in the neighbouring region, they broaden practically into large voids in the case of oversintered cermet. The latter is particularly evident in the case of Ni-SDC cermet sintered at 1400 °C. By contrast, the fuel gas is homogenously distributed at a certain propagation depth and equally falls along the Z-direction if the cermet is sintered at 1200 °C or 1100 °C due to an evenly distributed pore network.

Electrochemical and catalytic properties of Ni-SDC cermets toward fuel oxidation are normally described by the parameter TPBL, which is associated with the number of regions where the electrochemical reactions can occur. However, in addition to the fuel electrochemical oxidation itself, numerous additional reaction steps (i.e., adsorption and dissociation of hydrogen or hydrocarbon reforming within an anode layer of a SOFC), not necessarily located at the TPBs, must take place in an operating electrode. Therefore, besides the overall specific surface areas of individual phases (SSA), specific interface areas (SIA) of two adjacent phases are also very important second-order microstructural parameters. Both the SSA and SIA parameters will influence the surface chemical reactions and heat transfer in porous electrodes. The larger the parameter, the larger the surface will be exposed within the porous electrode, which may lead to a higher rate of several processes in an operating fuel cell. With respect to the previously discussed connectivity results, SSAs and SIAs are calculated only inside the percolated volume (Table 1) since isolated clusters may not contribute to the electrochemical performance of the cermets. For the investigated Ni-SDC cermets, the SSA values are rather high compared to some data published in the literature; however, they are obtained through the precise 3D microstructure analysis (small voxel size in relative large analysed volume) and are a consequence of the relatively small grains of each individual phase. The SSA values for pores decrease with increasing sintering temperature due to the fact that cermets exhibit less and less porosity with some closed pores if the sintering temperature rises. The SSA values of the two solid phases (Ni and SDC) are also expected to decrease with increasing sintering temperature owing to grain growth, with one exception. Specifically, as observed through the grain size analysis (feret values), some Ni enlargement during the NiO to Ni reduction is observed in the sample sintered at 1100 °C due to the non-rigid SDC framework. Consequently, this anomaly is expressed through the relatively low SSANi value when samples sintered at 1100 °C and 1200 °C are compared. No such effect is noticed for SSASDC values.

**Figure 7:**
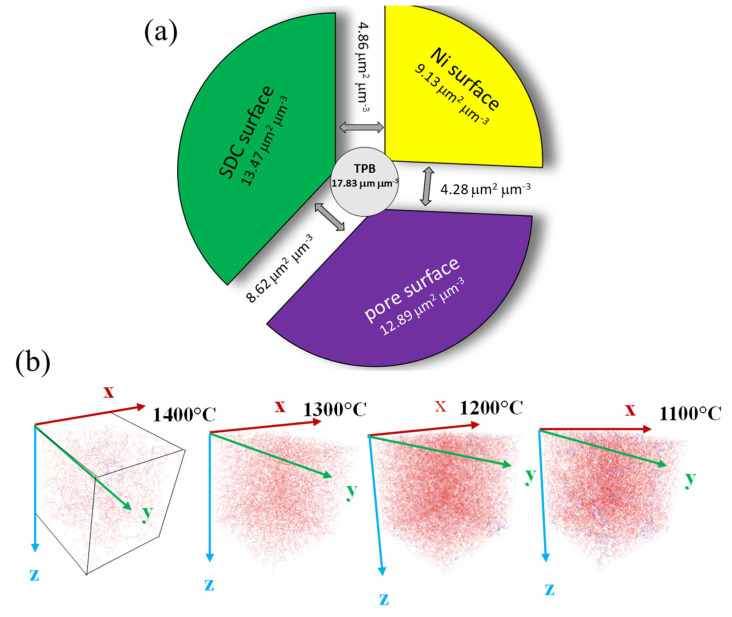
(**a**) Pie diagrams representing the SSAs of pore, Ni, and SDC phases and the SIAs between the three neighbouring phases for the Ni-SDC cermet sintered at 1200 °C. The active TPBL is written in the middle circle, and (**b**) visualization of TPBs (active coloured red and inactive coloured blue) in Ni-SDC cermets prepared under various sintering conditions.

Furthermore, each one-phase region in the cermet is in contact with the two adjacent one-phase regions, meaning that by a simple additional step in 3D image analysis during the SSA values determination, SIA values (normalised to the sample volume) can be calculated (Table 1). According to the obtained SSA and SIA values, after sintering at 1400 °C, about 23.6% of the total Ni surface is exposed to the porous phase and could be used for the surface catalytic reaction with the fuel (in Figure 7a, the total SSA of the Ni phase 5.47 μm^2^ μm^−3^ is divided into Ni-pore SIA 1.29 μm^2^ μm^−3^ and Ni-SDC SIA 4.19 μm^2^ μm^−3^). The rest (76.5%) of the Ni surface is oriented toward SDC and, thus, available for electrochemical reactions at the Ni-SDC interphase. Similar calculations regarding the fraction of Ni-facing pores or SDC can also be calculated for other cermets. It appears that with decreasing sintering temperature, the Ni-pore-to-Ni-SDC ratio varies among 0.31, 0.51, 0.88, and 0.78 if cermets are sintered at 1400 °C, 1300 °C, 1200 °C, or 1100 °C, respectively. Again, the anomaly in the increasing trend is observed for the Ni-SDC cermet sintered at 1100 °C, as mentioned previously, mainly due to the excessive Ni growth.

The electrode electrochemical performance can be directly linked with the TPB_L_. Therefore, the determination and visualisation of the TPBs are one of the prime tasks of the 3D microstructural investigation. In this context, it also has to be mentioned that under a reducing environment, SDC is a mixed electronic–ionic conductor [55]. Therefore, the electrochemically active regions of Ni-SDC cermets are not strictly limited to TPBs but rather extended to their vicinity [56]. However, with respect to purely geometrical aspects of the prepared Ni-SDC cermets, these electrochemical issues are neglected. Volumetric TPB_L_ density values for each sample are given in Table 1. The TPB_L_ values are divided into total TPB_L_ and active TPB_L_. The term ‘active TPB_L_’ is a subject of numerous definitions in the literature [57,58]. In the present work, we adopted the definition of an active TPB_L_ if it comprises the three percolated phases (Ni, SDC, and porosity). The visualisation of TPBs is presented in Figure 7b. In general, smaller Ni-particle sizes are preferable for larger TPB_L_ density [59]. The highest total TPB_L_, 21.16 μm μm^−3^, or only active TPB_L_, 17.83 μm μm^−3^, is determined for the Ni-SDC cermet sintered at 1200 °C. The discrepancy between total and active TPB_L_ at 1200 °C is mainly because the solid phases do not percolate completely, particularly Ni. With increasing sintering temperature from 1200 °C toward 1400 °C, the TPB_L_ density decreases. This reduction in TPB_L_ is in accordance with increasing average particle sizes with sintering temperature and, thus, the lower number of interface contacts. While the solid phases almost completely percolate if sintered at 1400 °C, the limiting factor reducing active TPB_L_ is mainly pore percolation and the appearance of closed porosity. In contrast, a sintering temperature of 1100 °C again decreases the TPB_L_. The main reason for such observed TPB_L_ reduction is predominantly in Ni-coarsening during NiO to Ni reduction (due to already explained exaggerated Ni-growth) and lower connectivity of solid phases while pores percolate almost completely.

## 4. Conclusions

This work described a 3D FIB-SEM microstructural analysis of Ni-SDC anode cermets. The Ni-SDC samples were prepared with citrate–nitrate combustion synthesis, followed by milling, sintering at various temperatures, and NiO to Ni reduction. The chosen synthesis route ensured a nano-sized starting NiO-SDC mixture (prior to sintering and reduction). The preparation of final Ni-SDC samples for FIB-SEM analysis was optimised to obtain high-quality representative 3D microstructures in which the total reconstructed volume in each case included at least 104 particles of a respective phase. In-house developed software rendered feasible the calculation of the desired microstructural parameters, i.e., volume fractions and particle size distribution of various phases, connectivity of phases, three-phase boundary length (TPBL), interfacial areas, tortuosity as well as absolute permeability and simulations of gas pressure drop during gas flow through the individual cermet. These calculations demonstrated that after the sintering, Ni and SDC phases remain well in the sub-micrometre range. Generally, a lower sintering temperature resulted in smaller individual grains. However, due to the insufficient sintering of the SDC phase at 1100 °C, excessive Ni growth was noticed. In contrast, well-sintered Ni-SDC cermet at 1400 °C retained only 18.9% porosity, of which 23.8% is closed porosity. Relatively large differences in the cermets’ porosities, if they were sintered at various temperatures, were also reflected through rather big differences in their absolute permeabilities. Interestingly, cermet sintered at 1400 °C contains relatively large regions, which are practically impermeable to gases. This further implies that such oversintered Ni-SDC cermets not only exhibited insufficient overall open porosity but were also locally inhomogeneous (regarding the same parameter). However, cermet sintered at 1200 °C exhibited homogeneous pathway distribution for gas transport, meaning that the majority of reactive centres can be effectively involved in electro-catalytic reactions. One of the prime tasks of the 3D microstructural investigation is the determination and visualisation of the TPBLs. The TPBL values were divided into total TPBL and active TPBL. Only active TPBL provides essential information related to the anode electrochemical activity. Advanced 3D microstructural analysis revealed that the highest active TPBL was achieved in the case of Ni-SDC cermet sintered at 1200 °C. Sintering at lower temperatures reduced the active TPBL due to insufficient connectivity (especially Ni-phase). However, sintering at higher temperatures reduced the active TPBL due to a more closed porosity and, thus, reduced SIA of Ni-pore and SDC-pore values.

## Figures and Tables

**Table 1:** Determined morphological and microstructural parameters of Ni-SDC cermets determined by 3D approach.

T_sintering_/°C			1100	1200	1300	1400	
Analysed volume (xyz)/μm^3^			6 × 6 × 6	6 × 6 × 6	8 × 8 × 8	12 × 12 × 12	
Voxel size (xyz)/nm^3^			4 × 4 × 8	4 × 4 × 8	5 × 5 × 10	10 × 10 × 10	
w_vol. Ni_/%			27.3	29.1	31.7	36.8	1st order parameters
w_vol. SDC_/%			33.0	35.3	39.0	44.3	
*feret/*μm	*x*	Ni	0.45	0.28	0.44	0.71	
		SDC	0.21	0.22	0.38	0.61	
	*y*	Ni	0.44	0.27	0.43	0.68	
		SDC	0.20	0.21	0.36	0.60	
	*z*	Ni	0.43	0.27	0.41	0.71	
		SDC	0.20	0.22	0.37	0.63	
$\bar{d}$/μm	Ni		0.46	0.28	0.45	0.72	
	SDC		0.21	0.23	0.38	0.64	
*Ψ_W_*/	Ni		0.71	0.82	0.82	0.79	
	SDC		0.89	0.87	0.86	0.85	
*ε*/%	microstructural		39.7	35.6	29.3	18.9	
*ε*/%	open		39.5	34.0	26.2	14.4	2nd order parameters
	closed		0.2	1.6	3.1	4.5	
Connectivity/%	Ni		69.4	89.2	98.7	99.8	
	SDC		89.0	94.8	99.9	100.0	
	pores		99.8	98.3	91.4	76.5	
TPB density/μm μm^−3^	total		18.34	21.16	9.51	4.71	
	active		11.09	17.83	8.93	3.76	
SSA/μm^2^ μm^−3^	Ni		7.24	9.14	7.09	5.48	
	SDC		15.01	13.48	10.73	7.21	
	pore		14.11	12.90	8.44	4.31	
SIA/μm^2^ μm^−3^	Ni-pore		3.17	4.28	2.40	1.29	
	Ni-SDC		4.07	4.86	4.69	4.19	
	SDC-pore		10.94	8.62	6.04	3.02	

**Table 2:** Geometrical tortuosity (*τ*) for various Ni-SDC cermets.

T_sint_/°C	1100			1200			1300			1400		
*τ_factor+geo_*	*τ_x_*	*τ_y_*	*τ_z_*	*τ_x_*	*τ_y_*	*τ_z_*	*τ_x_*	*τ_y_*	*τ_z_*	*τ_x_*	*τ_y_*	*τ_z_*
Ni	2.81	2.81	2.83	2.23	2.21	2.24	1.94	1.94	1.96	1.72	1.73	1.73
SDC	2.08	2.09	2.10	1.94	1.90	1.96	1.73	1.74	1.73	1.53	1.54	1.59
pores	1.23	1.23	1.25	1.28	1.29	1.28	1.54	1.54	1.53	2.49	2.50	2.49

## Data Availability

The original contributions presented in the study are included in the article/Supplementary Material, further inquiries can be directed to the corresponding author.

## Supplementary Materials

The following supporting information can be downloaded at: https://www.mdpi.com/article/10.3390/ma17133068/s1, Figure S1: (a) Thinned FIB-SEM samples attached to Al-stub; (b) detail with enlarged lamella; Figure S2: (a) Deposited Pt-layer on ROI with shortly milled lines; (b) deposition of C on milled lines; (c) secondary Pt-deposition; (d) cross-section of ROI with reference markers; Figure S3: (a) raw image data volume, (b) image data volume after direct 3D image processing. Half orthogonal plane dimension, marked with the yellow arrow on the YZ plane, corresponds to 1.5 mm (below); Figure S4: Three-dimensional particle separation within examined Ni-SDC cermet: (a–d) particle separation procedure demonstrated on a 2D projection; (e) continuously connected SDC phase and (f) three-dimensionally separated particles within the SDC volume; Figure S5: AR-STEM EDXS analysis of NiO-SDC initial nanosized powder; Figure S6: Electron diffraction on NiO-SDC initial nanosized powder; Figure S7: Particle size distribution of ungrounded powder, powder after 1st milling cycle and after 2nd milling cycle.
