# Supplementary material for: The Microstructural Reconstruction of Variously Sintered Ni-SDC Cermets Using Focused Ion Beam Scanning Electron Microscopy Nanotomography
Source: Materials (Basel). 2024 Jun 21;17(13):3068. doi: 10.3390/ma17133068 (PMC11242238; doi:10.3390/ma17133068)
Supplement: Supplementary file 1 [file materials-17-03068-s001.zip › supplementary figures.pdf]

Supplementary Figures

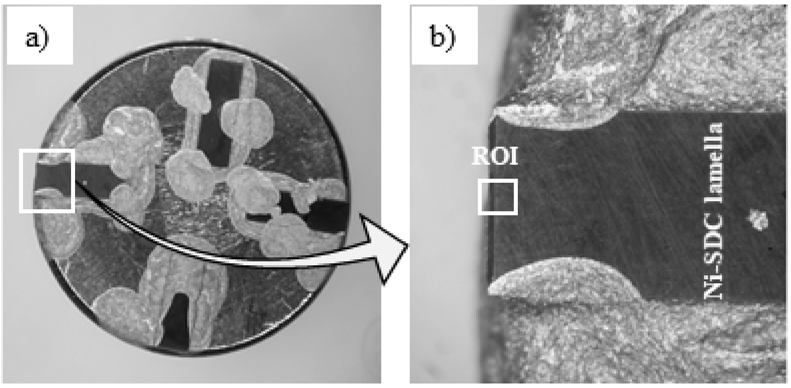

Figure S1. (a) Thinned FIB-SEM samples attached to Al-stub; (b) detail with enlarged lamella.

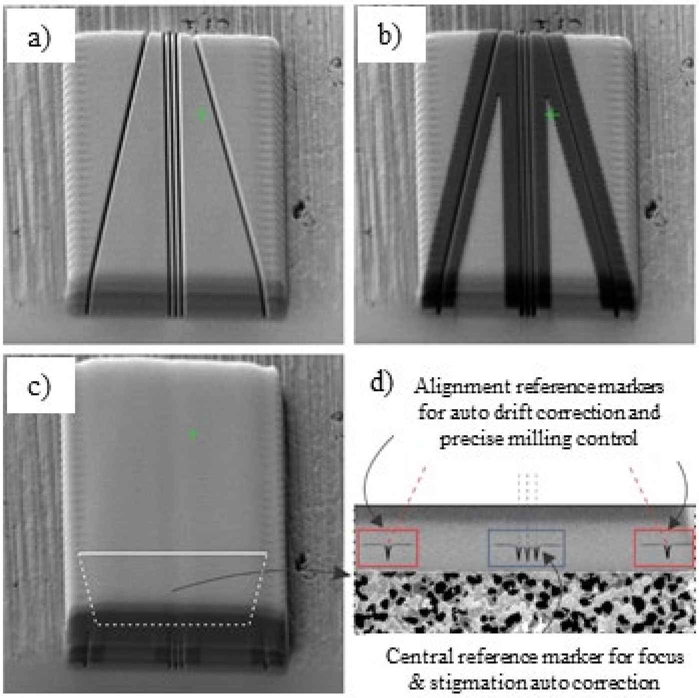

Figure S2. (a) Deposited Pt-layer on ROI with shortly milled lines; (b) deposition of C on milled lines; (c) secondary Pt-deposition; (d) cross-section of ROI with reference markers.

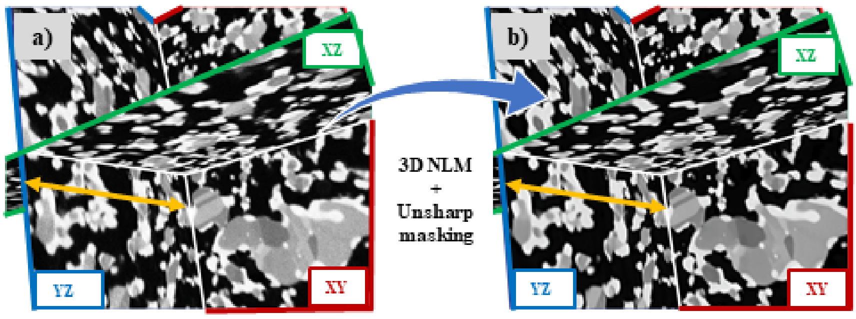

Figure S3. (a) raw image data volume, (b) image data volume after direct 3D image processing. Half orthogonal plane dimension, marked with the yellow arrow on the YZ plane, corresponds to 1.5 mm (below.

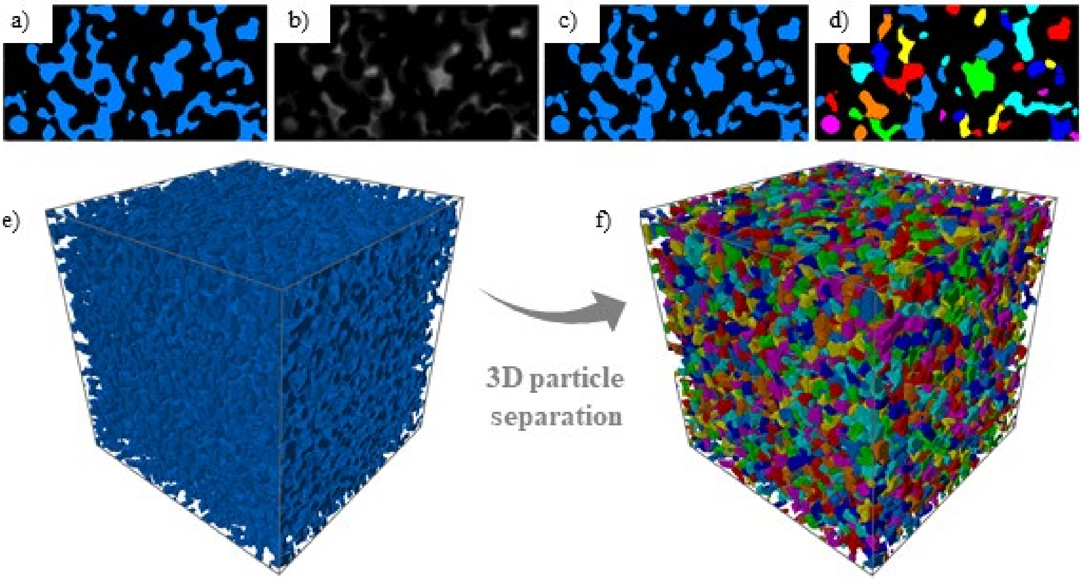

Figure S4. Three-dimensional particle separation within examined Ni-SDC cermet: (a–d) particle separation procedure demonstrated on a 2D projection; (e) continuously connected SDC phase and (f) three-dimensionally separated particles within the SDC volume.

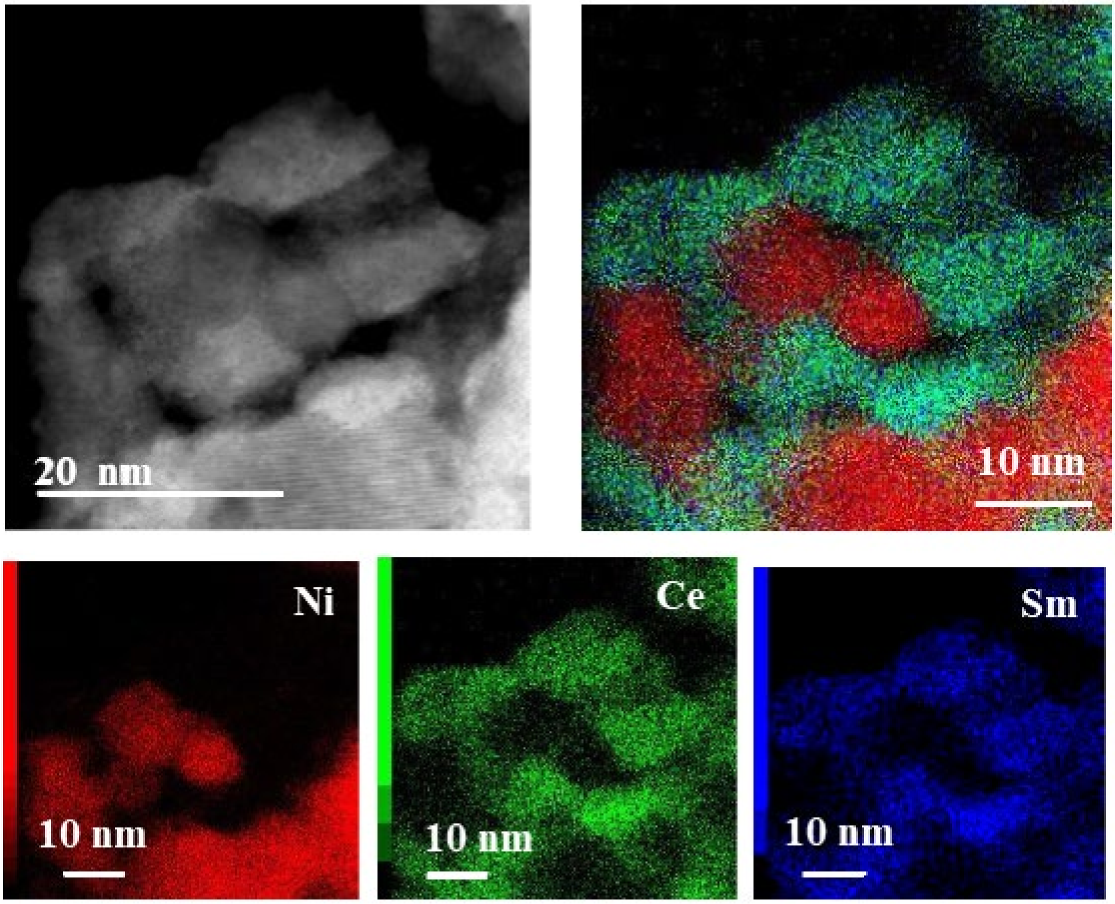

Figure S5. AR-STEM EDXS analysis of NiO-SDC initial nanosized powder.

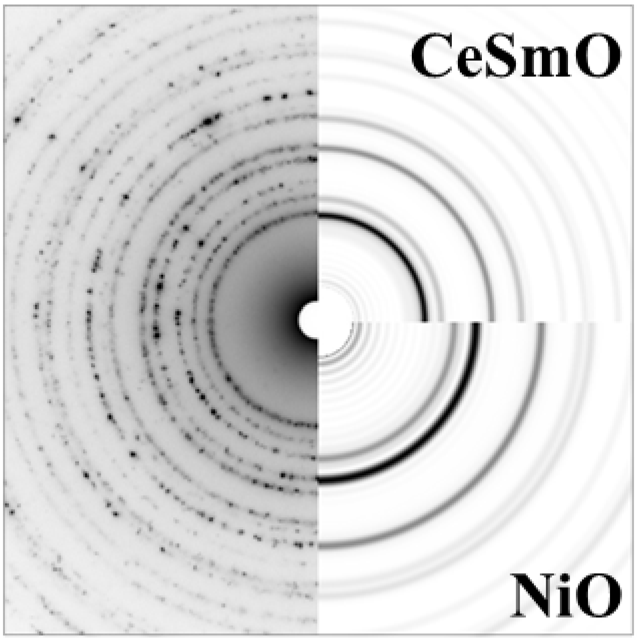

Figure S6. Electron diffraction on NiO-SDC initial nanosized powder.

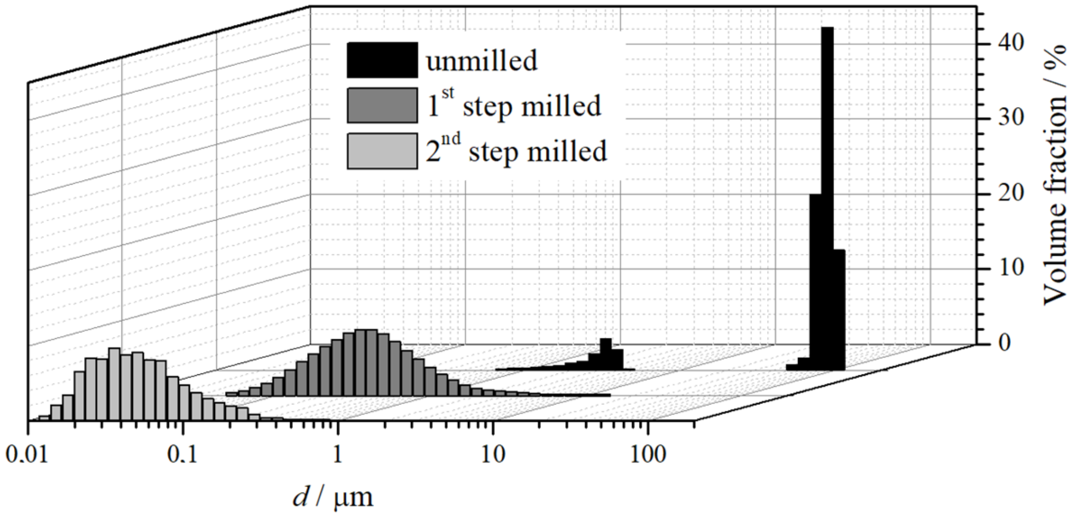

Figure S7. Particle size distribution of ungrounded powder, powder after 1st milling cycle and after 2nd milling cycle.
